# Supplementary material for: Examining the thermotropic properties of large circularized nanodiscs
Source: Biochim Biophys Acta Biomembr. Author manuscript; Available in PMC 2025 Nov 3. (PMC12580977; doi:10.1016/j.bbamem.2025.184451)
Supplement: Supplementary [file NIHMS2119502-supplement-Supplementary.pdf]

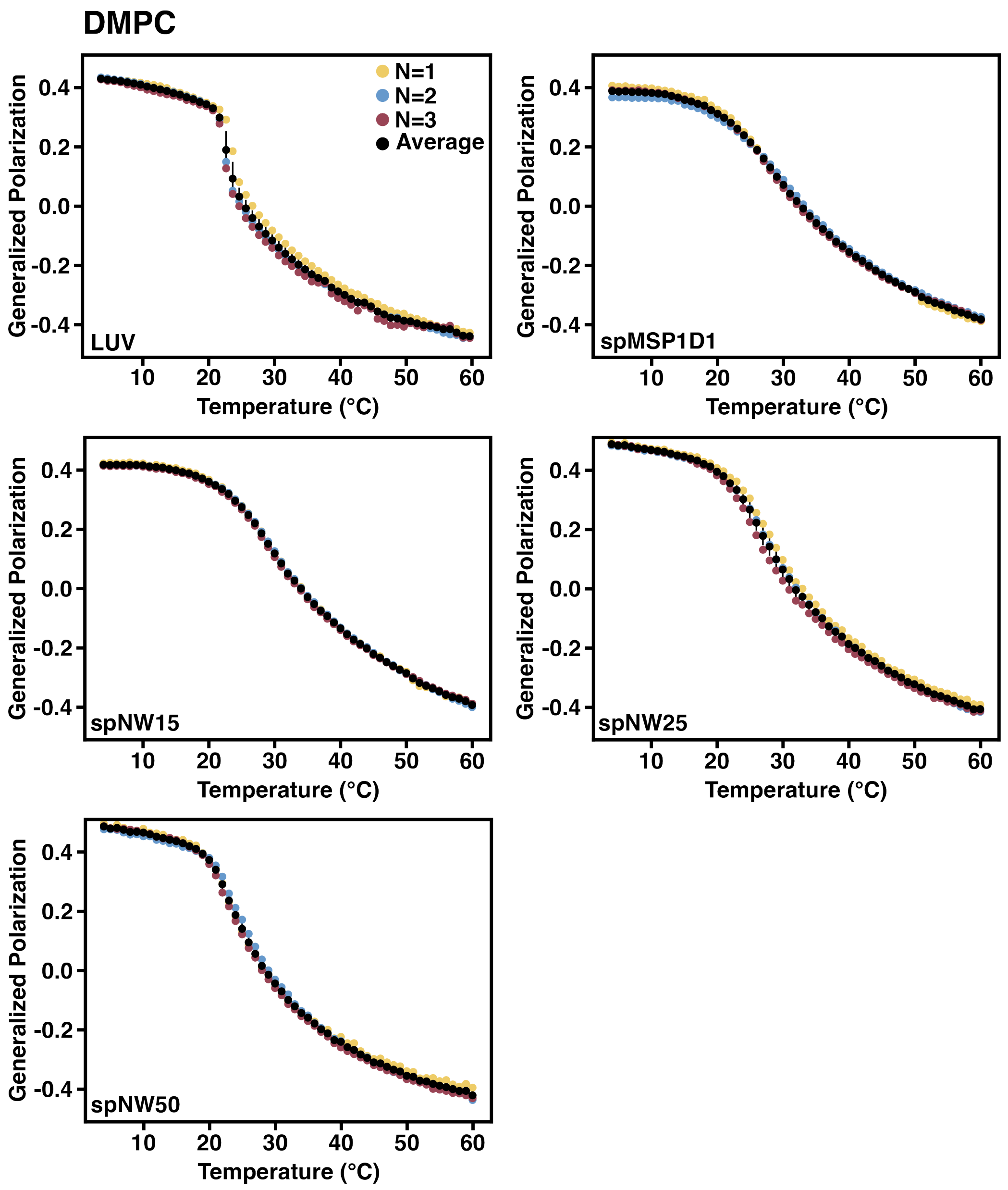

Figure S1: Generalized polarization versus temperature for all three independent DMPC replicates (yellow, blue, and red dots). Average across the three independent replicates is shown as black dots with the error bar representing the standard deviation across the three replicates.

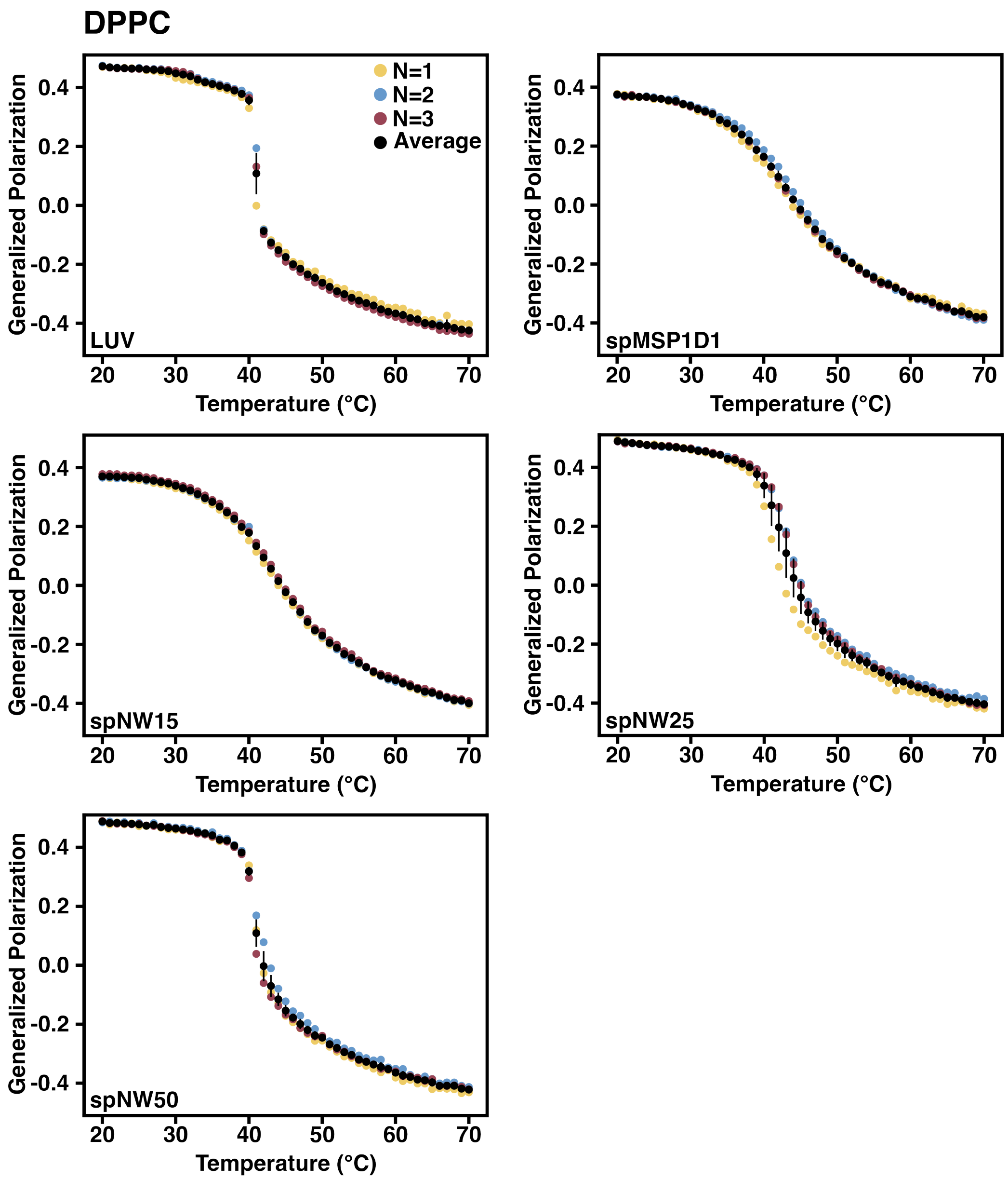

Figure S2: Generalized polarization versus temperature for all three independent DPPC replicates (yellow, blue, and red dots). Average across the three independent replicates is shown as black dots with the error bar representing the standard deviation across the three replicates.

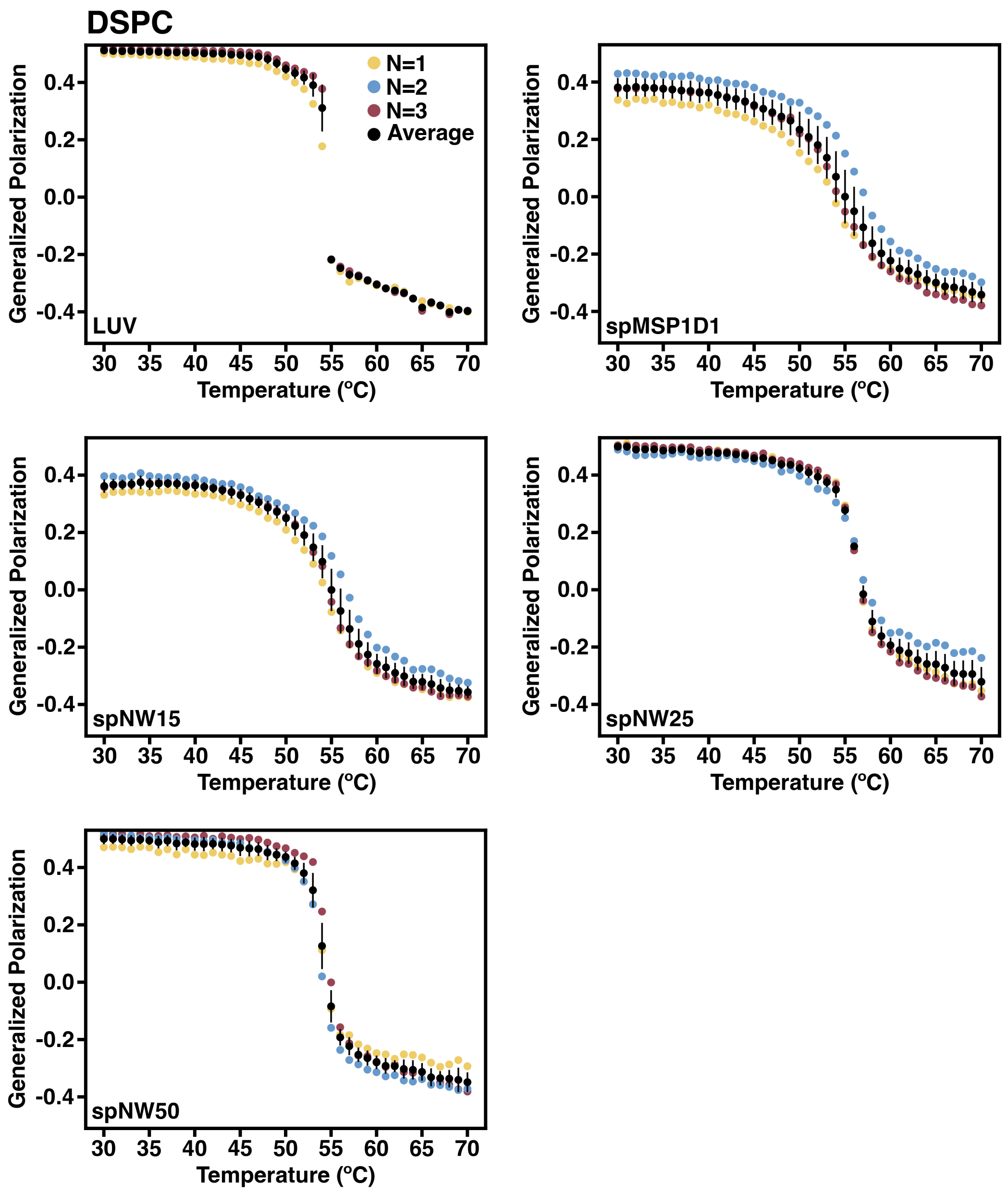

Figure S3: Generalized polarization versus temperature for all three independent DPPC replicates (yellow, blue, and red dots). Average across the three independent replicates is shown as black dots with the error bar representing the standard deviation across the three replicates.

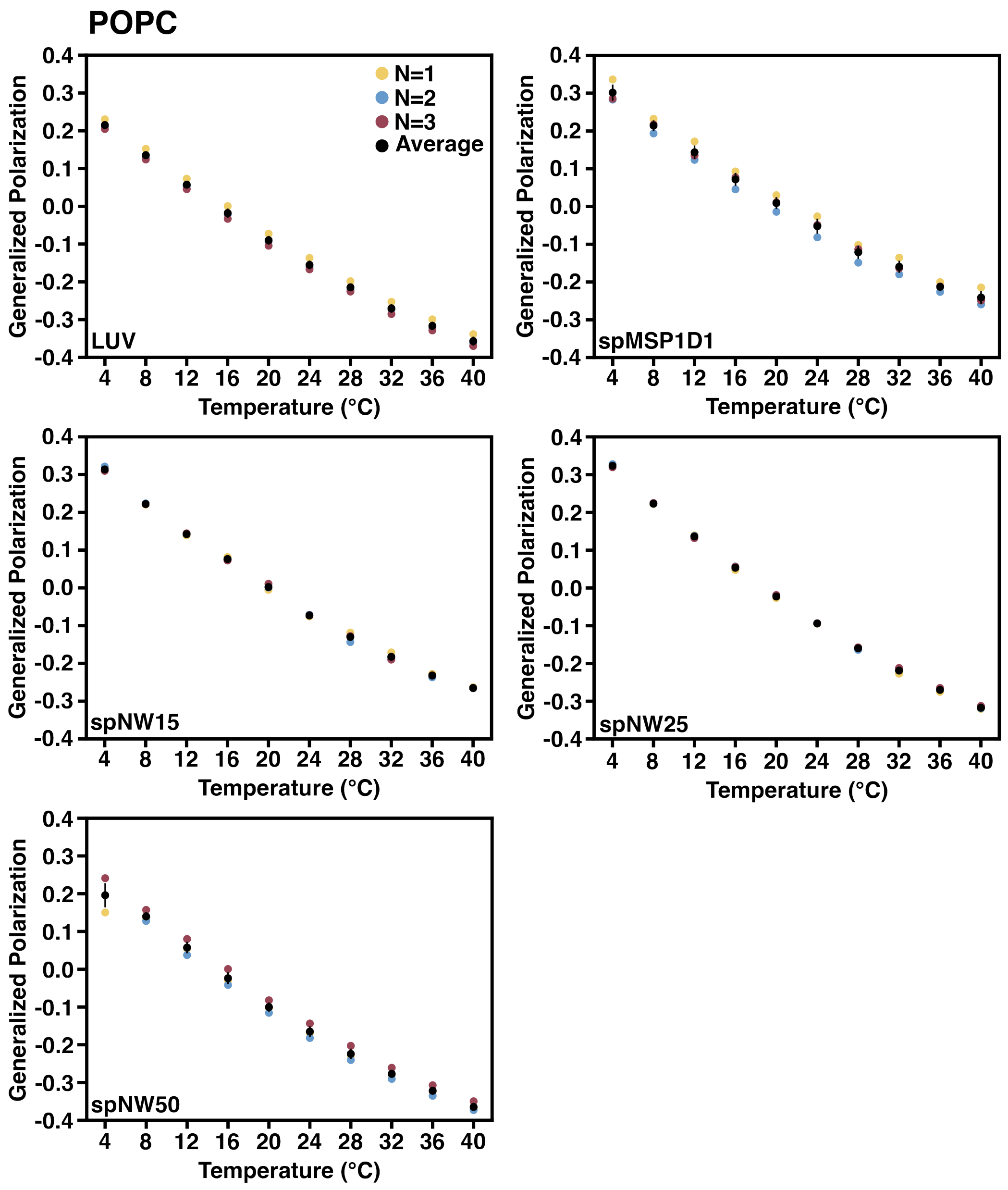

Figure S4: Generalized polarization versus temperature for all three independent POPC replicates (yellow, blue, and red dots). Average across the three independent replicates is shown as black dots with the error bar representing the standard deviation across the three replicates.

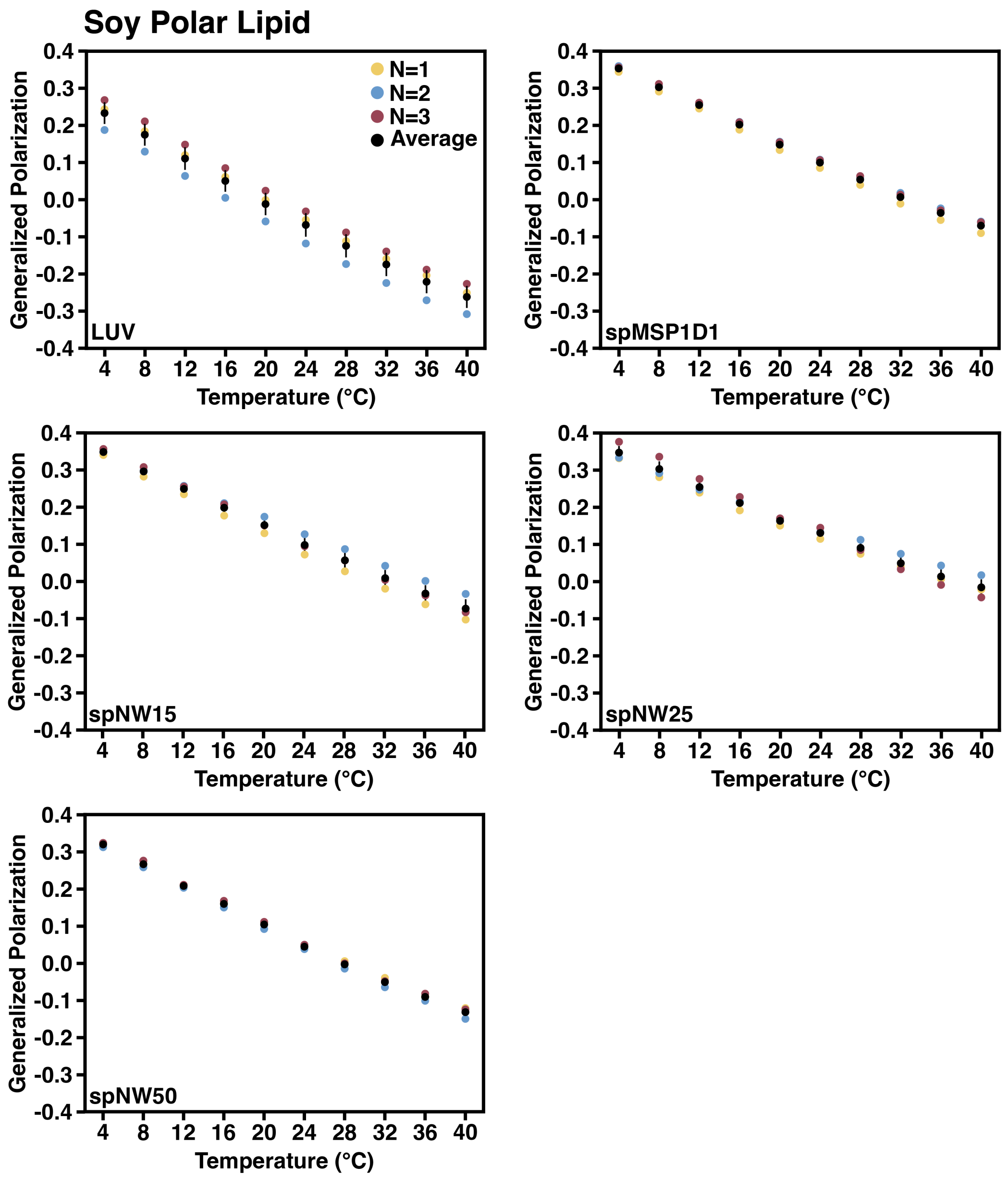

Figure S5: Generalized polarization versus temperature for all three independent soy polar extract replicates (yellow, blue, and red dots). Average across the three independent replicates is shown as black dots with the error bar representing the standard deviation across the three replicates.

Lipid		Nanodisc (spNW15)		Control	
		Rel. Abundance (%)	Std. Dev.	Rel. Abundance (%)	Std. Dev.
PC	32:0	0.395	0.064	0.373	0.114
	32:1	0.119	0.144	0.136	0.029
	34:1	3.114	0.073	2.989	0.034
	34:2	54.291	0.061	53.326	0.029
	34:3	4.845	0.057	4.725	0.026
	35:3	1.618	0.057	1.209	0.002
	36:2	14.302	0.058	13.771	0.018
	36:3	21.245	0.067	20.826	0.034
	36:4	100.000	0.055	100.000	0.036
	36:5	25.873	0.041	20.856	0.021
	37:4	16.662	0.170	5.592	0.075
	37:5	2.815	0.166	1.041	0.088
	37:6	0.166	0.141	0.044	0.025
	38:2	2.195	0.153	0.801	0.061
	38:6	3.338	0.164	1.148	0.091
	39:2	0.293	0.085	0.270	0.031
	39:3	0.310	0.036	0.2996	0.0002
	39:4	0.274	0.028	0.215	0.034
	40:2	—	—	0.079	0.083
	40:3	0.042	0.281	0.059	0.490
PE	lyso-16:0	0.045	0.043	0.109	0.012
	lyso-18:1	0.01	0.19	0.481	0.005
	lyso-18:2	0.28	0.12	0.303	0.042
	32:0	0.04	0.01	0.025	0.011
	34:1	0.52	0.21	0.531	0.065
	34:2	8.186	3.233	8.186	0.994
	34:3	0.576	0.246	0.624	0.065
	36:2	0.87	0.36	0.911	0.103
	36:3	1.435	0.613	1.552	0.191
	36:4	8.38	3.74	9.46	1.01
	36:5	1.438	0.676	1.71	0.17
	lyso-16:0	—	—	0.21	0.01
	lyso-18:0	—	—	0.013	0.005
	lyso-18:1	—	—	0.066	0.011

	lyso-18:2	—	—	0.653	0.015
	lyso-18:3	—	—	0.056	0.005
	32:0	0.016	0.008	0.016	0.011
	32:1	0.02	0.01	0.01	0.00
	33:2	0.015	0.005	0.006	0.005
	34:1	0.576	0.091	0.463	0.023
	34:2	9.73	1.61	8.346	0.333
	34:3	0.828	0.147	0.74	0.03
PA	35:2	0.151	0.011	0.11	0.01
	36:1	0.09	0.01	0.07	0.00
	36:2	1.89	0.30	1.48	0.03
	36:3	3.09	0.51	2.59	0.08
	36:4	17.23	2.71	15.54	0.22
	36:5	3.32	0.59	2.85	0.07
	36:6	0.18	0.03	0.17	0.01
	37:4	0.161	0.011	0.11	0.00
	38:2	0.07	0.05	0.01	0.01
	38:3	0.06	0.05	0.01	0.01
	38:4	0.178	0.014	0.125	0.007
	40:2	0.245	0.046	0.156	0.021
	40:5	0.46	0.07	0.06	0.03
	41:2	0.08	0.02	0.045	0.021
	42:2	0.14	0.02	0.076	0.005
	lyso-16:0	0.02	0.01	2.015	0.106
	lyso-18:0	—	—	0.33	0.01
	lyso-18:1	0.01	0.00	0.20	0.01
	lyso-18:2	0.02	0.01	1.84	0.04
	lyso-18:3	—	—	0.20	0.00
	32:0	0.114	0.023	0.125	0.007
	32:1	0.056	0.008	0.07	0.00
	32:2	0.081	0.007	0.10	0.00
	33:0	0.043	0.013	0.03	0.00
	33:1	0.05	0.01	0.04	0.01
	33:2	0.258	0.009	0.26	0.00
	33:3	0.09	0.01	0.04	0.00
PI	34:2	100.00	0.00	100.00	0.00
	34:3	13.465	0.144	13.97	0.09

	34:4	0.024	0.008	0.05	0.01
	35:2	0.648	0.023	0.655	0.021
	35:3	0.16	0.01	0.17	0.00
	36:2	19.695	0.212	19.62	0.07
	36:3	9.528	0.110	10.07	0.24
	36:4	15.495	0.118	15.555	0.176
	36:5	2.955	0.066	2.97	0.05
	36:6	0.218	0.004	0.215	0.007
	38:2	0.025	0.005	0.025	0.007
	40:2	0.196	0.005	0.18	0.01
PG	lyso-16:0	—	—	0.32	0.02
	lyso-18:0	—	—	0.01	0.00
	lyso-18:1	—	—	0.02	0.01
	lyso-18:2	—	—	0.33	0.03
	30:1	0.012	0.008	0.01	0.00
	31:1	0.033	0.008	0.01	0.00
	31:2	0.161	0.029	0.075	0.005
	31:3	0.03	0.00	0.01	0.00
	32:0	1.105	0.043	1.052	0.011
	32:1	0.545	0.021	0.044	0.005
	32:2	0.078	0.014	0.017	0.005
	33:1	0.220	0.008	0.01	0.00
	33:2	0.143	0.012	0.097	0.011
	33:3	0.238	0.027	0.260	0.015
	33:4	0.551	0.079	0.26	0.02
	33:5	0.148	0.033	0.057	0.015
	34:1	2.87	0.07	0.00	0.00
	34:2	35.62	1.02	35.61	0.41
	34:3	2.34	0.06	2.37	0.02
	35:1	0.071	0.014	—	—
	35:2	0.29	0.01	0.243	0.005
	35:5	0.13	0.01	0.194	0.005
	36:2	3.725	0.066	3.051	0.036
	36:3	1.741	0.057	1.68	0.05
	36:4	6.603	0.168	6.58	0.12
	36:5	1.005	0.032	1.03	0.01
	38:2	0.01	0.00	0.00	0.00

	40:0	0.102	0.012	0.17	0.02
	40:2	0.040	0.006	0.022	0.005

Table S1: Relative abundance of lipid species in nanodiscs and starting mixture. This table shows the relative abundance of each lipid type displayed by headgroup and tail length/unsaturation (length:unsaturation number) along with the standard deviation (n=3 for nanodisc, n=2 for control). A "—" signifies that particular lipid species was not detected in any of the samples.
